# Supplementary material for: Intratumor graph neural network recovers hidden prognostic value of multi-biomarker spatial heterogeneity
Source: Nat Commun. 2022 Jul 22;13:4250. doi: 10.1038/s41467-022-31771-w (PMC9307796; doi:10.1038/s41467-022-31771-w)
Supplement: Supplementary file 1 — Supplementary Information [file 41467_2022_31771_MOESM1_ESM.pdf]

Supplementary Information

Intratumor graph neural network recovers hidden prognostic value of multi-biomarker spatial heterogeneity

Qiu *et al.*

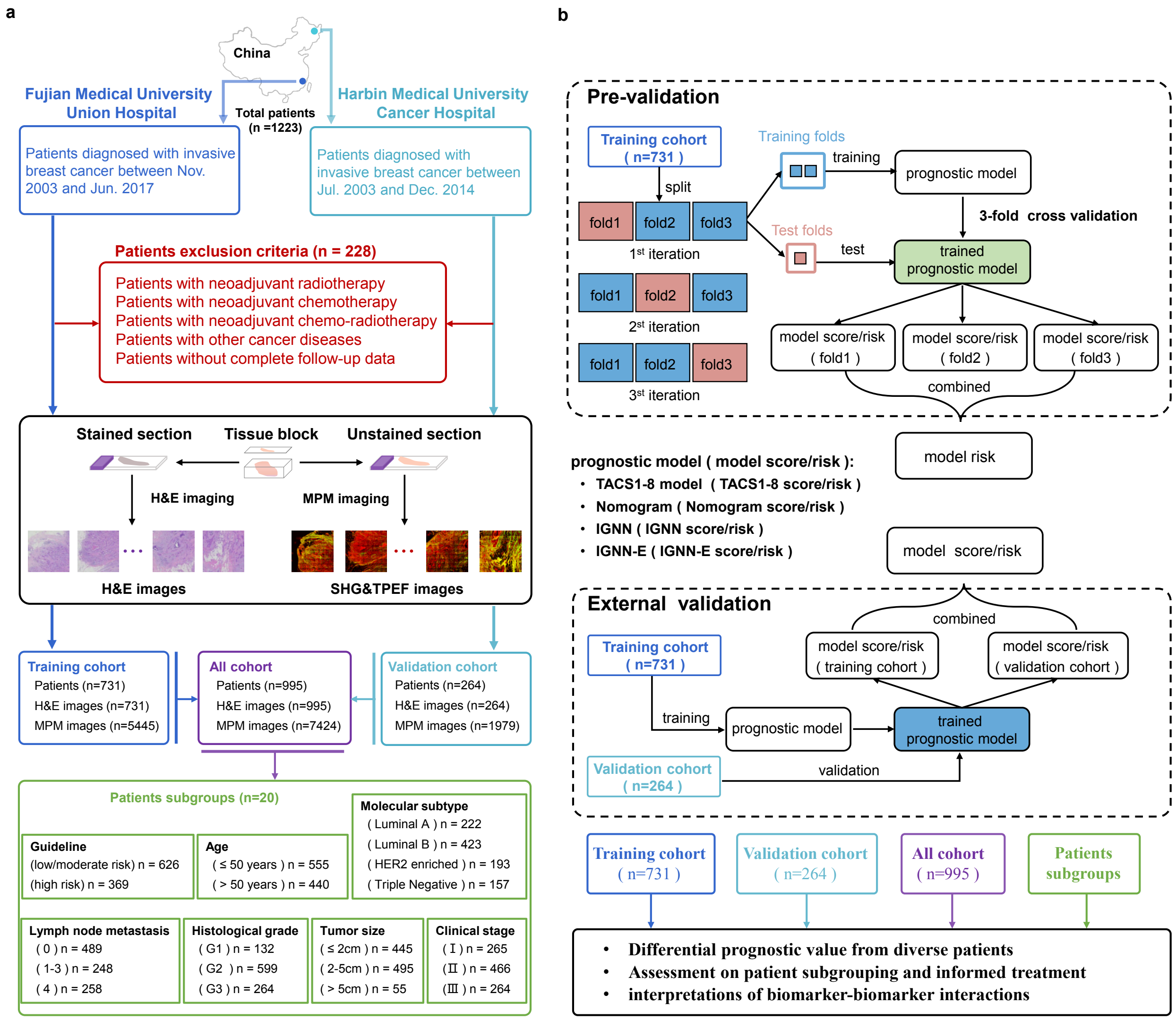

Supplementary Fig. 1. Overview of retrospective workflow. **(a)** Data of follow-up information and H&E/MPM images from Fujian Medical University Union Hospital (Fuzhou, China) and Harbin Medical University Cancer Hospital (Harbin, China). **(b)** Workflow demonstrating the construction and evaluation framework of prognostic models. The models were first constructed using pre-validation based on 3-fold cross validation within the training cohort to produce model score for each patient and assess their risk stratification capabilities. The resulting models were then retrained using the whole training cohort and finally applied to the validation cohort in an external validation process. H&E, hematoxylin and eosin; MPM, multiphoton microscopy; TPEF, two-photon excited (intrinsic) fluorescence; SHG, second harmonic generation; HER2, human epidermal growth factor receptor 2; TACS (TACS1-8), tumor-associated collagen signatures; Nomogram, extended model of multivariate Cox proportional hazard regression; IGNN, intratumor graph neural network; IGNN-E, extended IGNN model with clinical information. Source data are provided as a Source Data file.

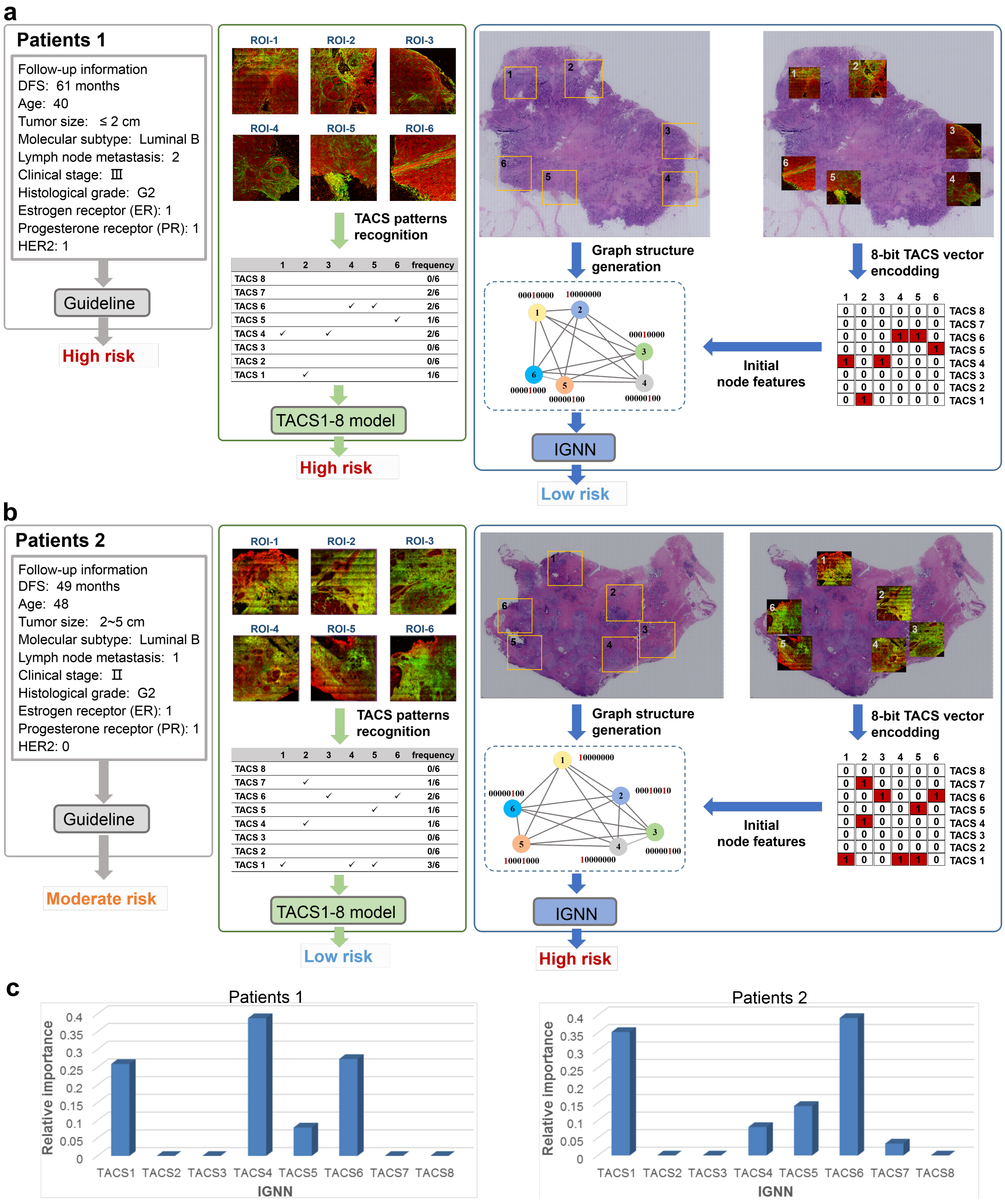

Supplementary Fig. 2. Examples demonstrating the unusual prognostic capability of the IGNN model. **(a)** A patient with DFS > 5 years but predicted to be at high risk by clinical treatment guideline and TACS1-8 model was predicted to be at low risk by the IGNN model. **(b)** A patient with DFS < 5 years but predicted to be at moderate or low risk by the guideline and TACS1-8 model was predicted to be at high risk by the IGNN model. **(c)** Attribution analysis based on the integrated gradient method that reveals the relative importance of different TACS features. DFS, disease-free survival; TACS (TACS1-8); HER2, human epidermal growth factor receptor 2; TACS (TACS1-8), tumor-associated collagen signatures; IGNN, intratumor graph neural network.

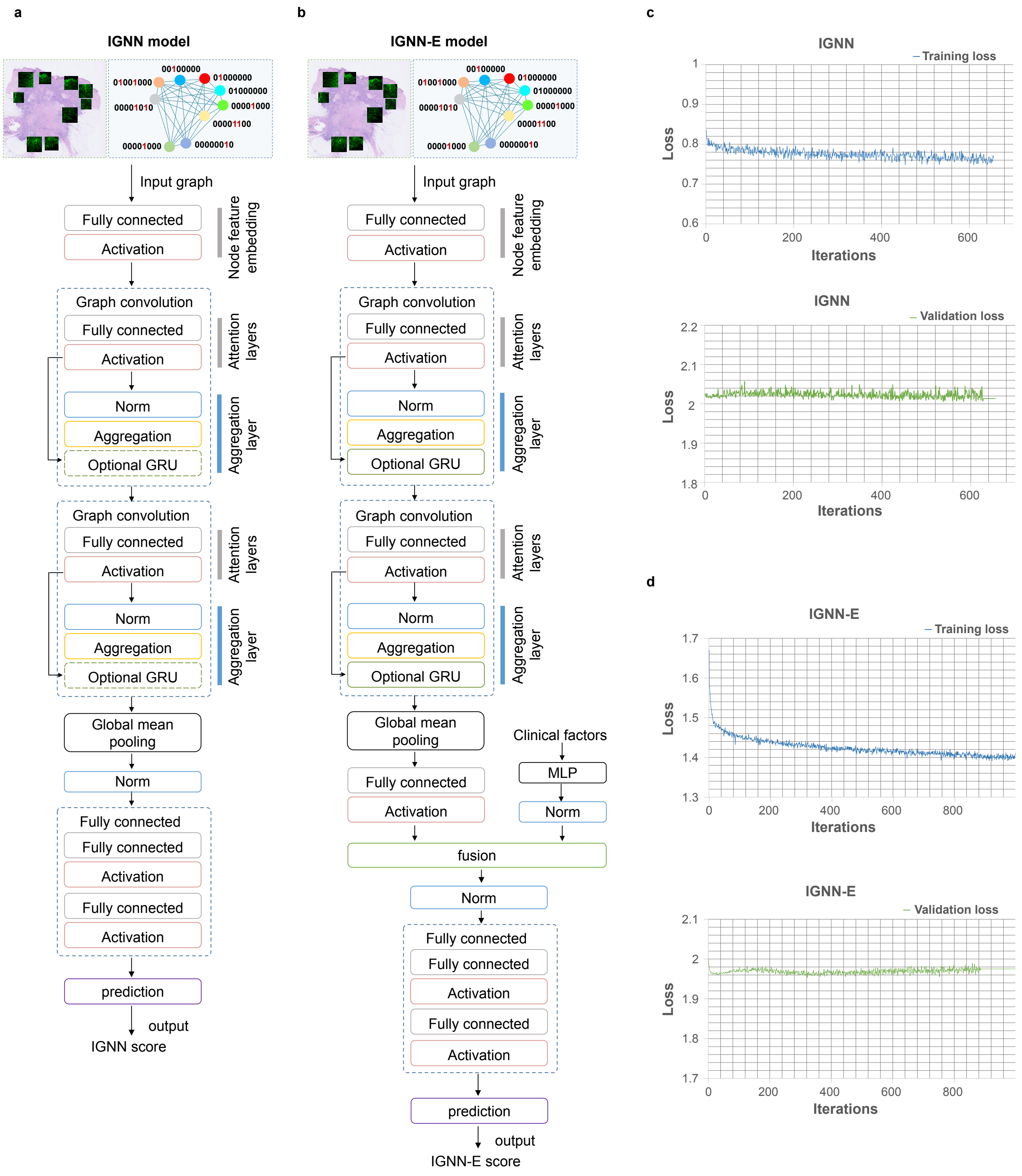

Supplementary Fig. 3. Architecture of two IGNN-based models. **(a)** IGNN model to predict prognosis from input graph structures. **(b)** IGNN-E model combining the IGNN model with additional MLP module and data fusion layer to predict prognosis from input graph structure data and clinicopathological factors (see Methods section). **(c, d)** Typical plots of the training and validation loss during the training of the two models. GRU, gated recurrent units; Norm, Normalization; MLP, Multi-layer perception machine; IGNN, intratumor graph neural network; IGNN-E, extended IGNN model with clinical information. Source data associated with **(c)** and **(d)** are provided as a Source Data file.

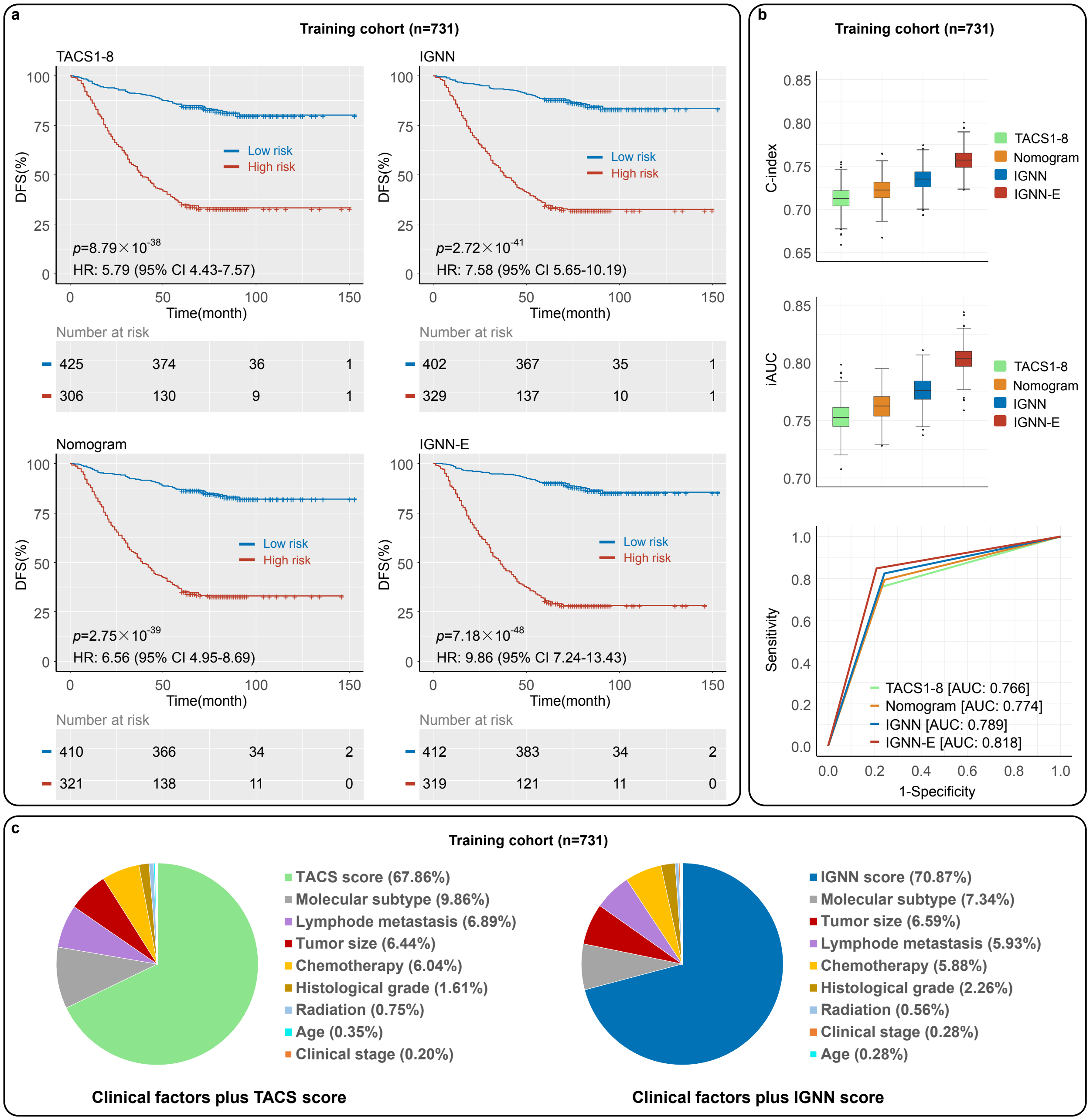

Supplementary Fig. 4. Performance of four prognostic models in pre-validation of training cohort (*n* is number of patients within the cohort). **(a)** Kaplan-Meier survival analysis of patients stratified into high risk group (red line) and low risk group (blue line) by different prognostic models (two-sided log-rank test to determine significance *p*). **(b)** Distribution of C-index (top) and iAUC panel (middle), and ROC curves and AUCs for 5-year DFS rate of prognostic risk predicted by different models (bottom). For boxplots illustrated within the C-index and iAUC panel, middle line represents the median value, the upper and lower boundaries of boxes indicate 25th and 75th percentile, the whiskers reflect 1.5 times of interquartile range, the upper and lower tails indicate the maxima and minima, and single points indicate the outliers, respectively. A two-sided unpaired t-test was performed to determine significance. **(c)** Relative contributions of prognostic biomarkers in predicting DFS in training cohort according to multivariate Cox proportional hazard regression analysis. Chi-squared test was performed to determine significance. DFS, disease-free survival; HR, hazard ratio; C-index, concordance index; iAUC, integrated cumulative/dynamic AUC; TACS (TACS1-8), tumor-associated collagen signatures; Nomogram, extended model of multivariate Cox proportional hazard regression; IGNN, intratumor graph neural network; IGNN-E, extended model by incorporating clinical information to the basic IGNN model. Source data are provided as a Source Data file.

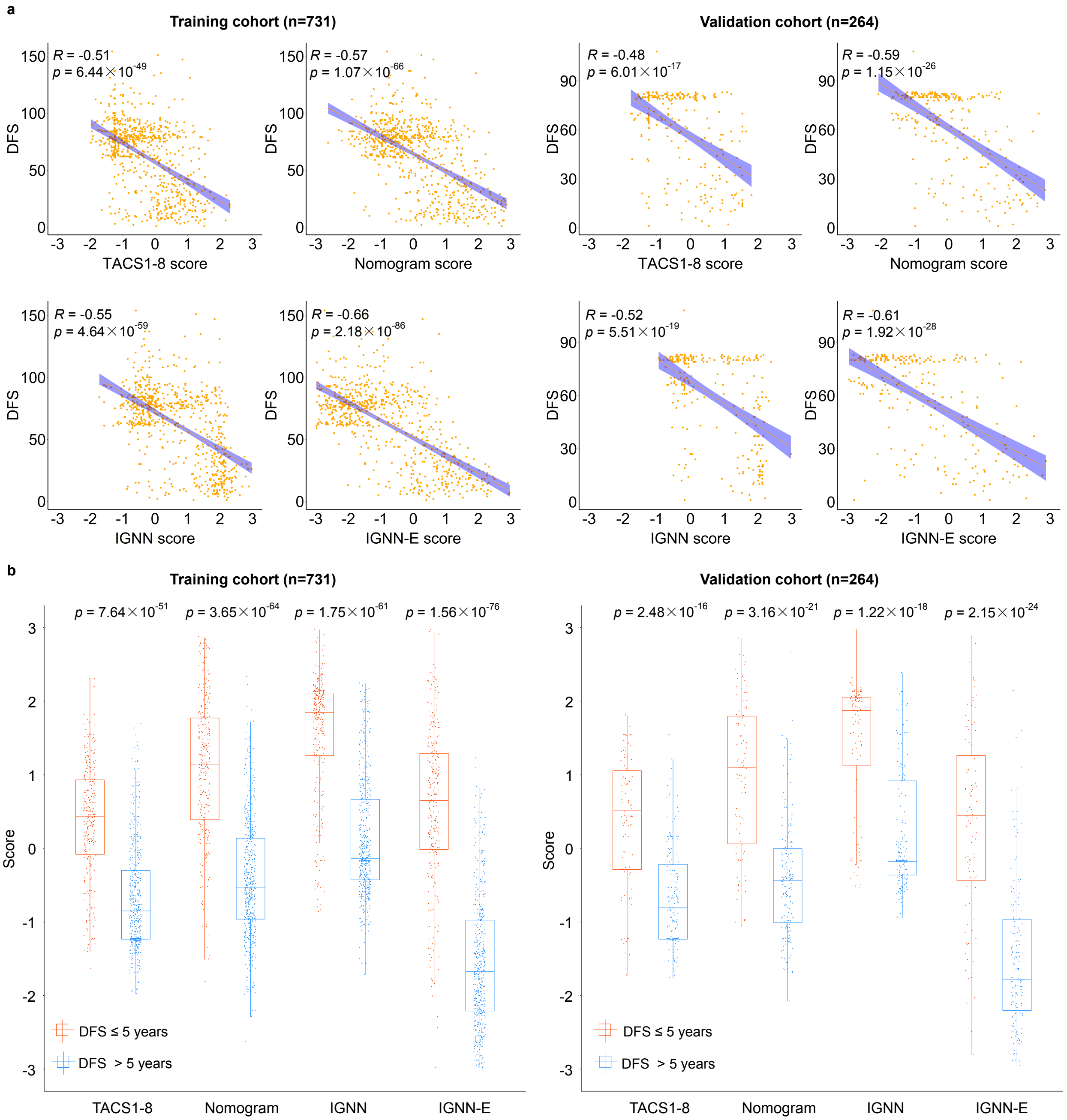

Supplementary Fig. 5. Correlation of model prediction scores with patient survival in training and validation cohorts (n is number of patients within the specified cohort). **(a)** Scatter plots of significant association between a model prediction score and DFS. Correlation analyses were performed using Pearson correlation. R statistic ranged from −1 to 1 indicates Pearson's product moment correlation coefficient (two-sided unpaired t-test was performed to determine significance p). **(b)** Corresponding boxplots showing the comparison between different prognostic model scores for patients with DFS less and more than 5 years. For boxplots, middle line represents the median value, the upper and lower boundaries of boxes indicate 25th and 75th percentile, the whiskers reflect 1.5 times of interquartile range, the upper and lower tails indicate the maxima and minima, and single points indicate the distribution of values. A two-sided unpaired t-test was used to compare risk subgroups and determine significance via exact p values. DFS, disease-free survival; TACS1-8, tumor-associated collagen signatures; Nomogram, extended model of multivariate Cox proportional hazard regression; IGNN, intratumor graph neural network; IGNN-E, extended IGNN model with clinical information. Source data are provided as a Source Data file.

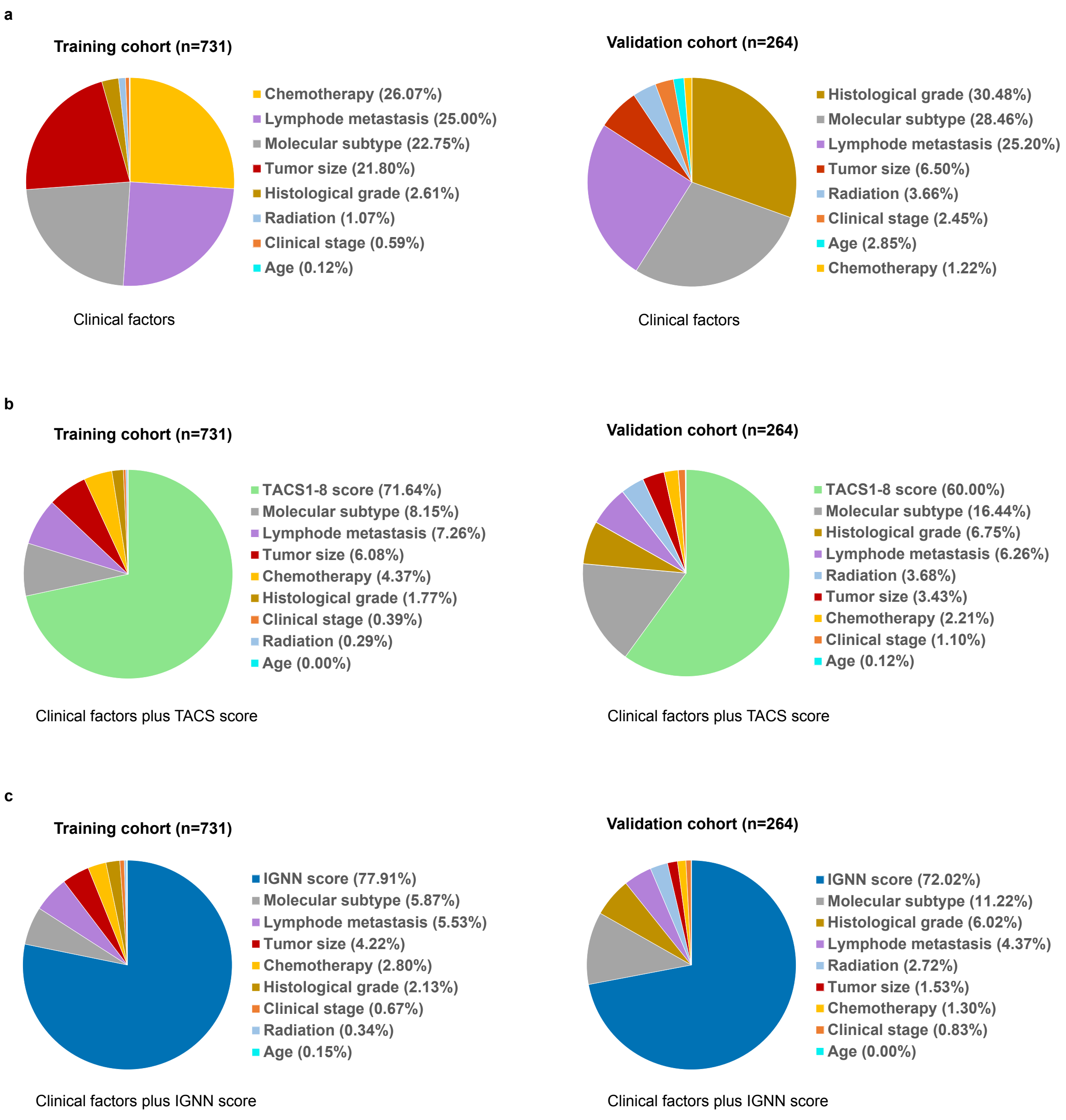

Supplementary Fig. 6. Relative contributions of various prognostic biomarkers in predicting DFS of 995 patients in external validation according to multivariate Cox proportional hazard regression analysis (*n* is number of patients within the specified cohort). **(a)** Routine prognostic biomarkers and TACS score **(b)** and routine prognostic biomarkers and IGNN score **(c)**. Chi-squared test was performed to determine significance. TACS (TACS1-8), tumor-associated collagen signatures; IGNN, intratumor graph neural network. Source data are provided as a Source Data file.

a

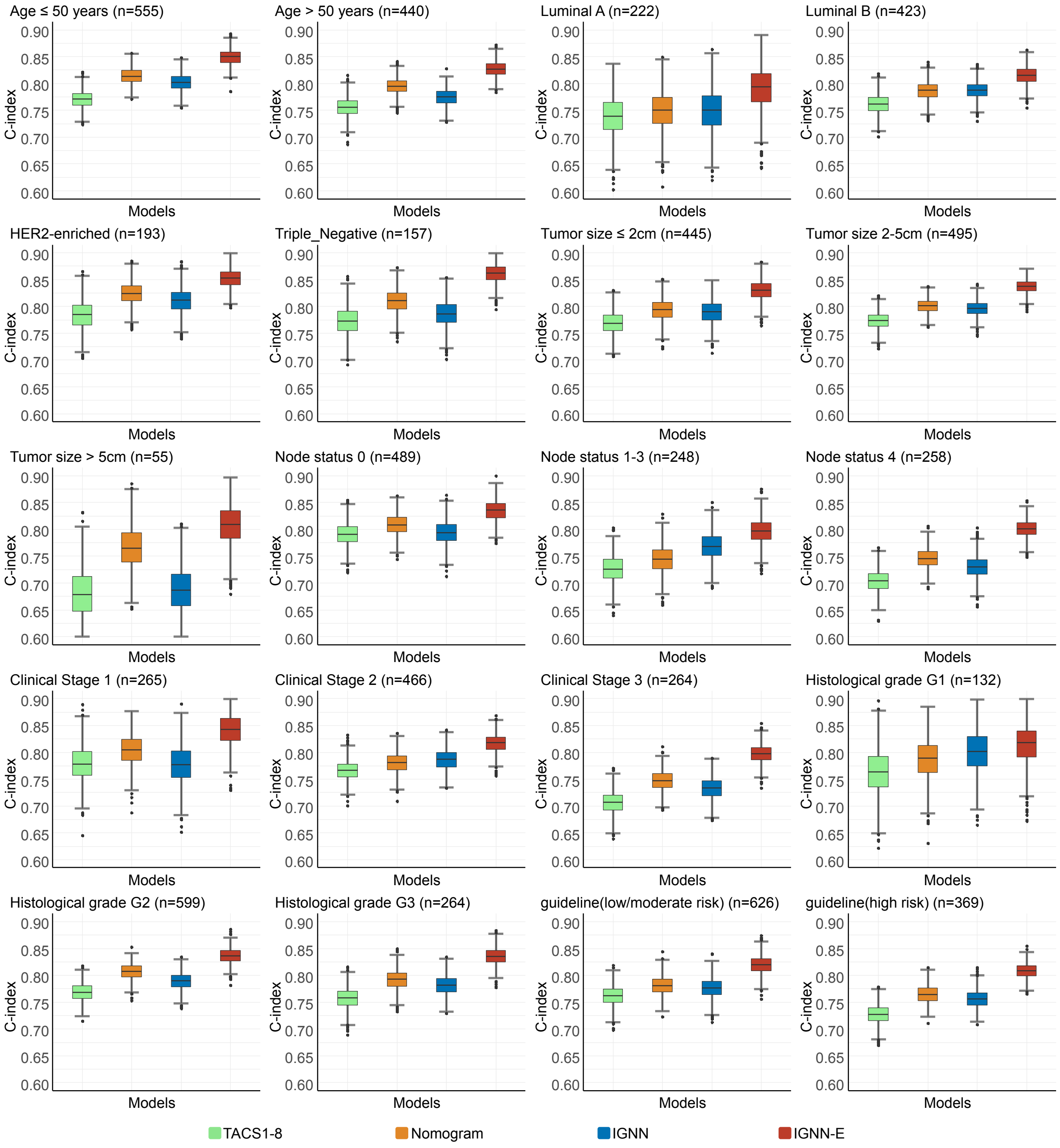
Age ≤ 50 years (n=555)
Age > 50 years (n=440)
Luminal A (n=222)
Luminal B (n=423)
HER2-enriched (n=193)
Triple_Negative (n=157)
Tumor size ≤ 2cm (n=445)
Tumor size 2-5cm (n=495)
Tumor size > 5cm (n=55)
Node status 0 (n=489)
Node status 1-3 (n=248)
Node status 4 (n=258)
Clinical Stage 1 (n=265)
Clinical Stage 2 (n=466)
Clinical Stage 3 (n=264)
Histological grade G1 (n=132)
Histological grade G2 (n=599)
Histological grade G3 (n=264)
guideline(low/moderate risk) (n=626)
guideline(high risk) (n=369)
C-index
0.60
0.65
0.70
0.75
0.80
0.85
0.90
Models
TACS1-8
Nomogram
IGNN
IGNN-E

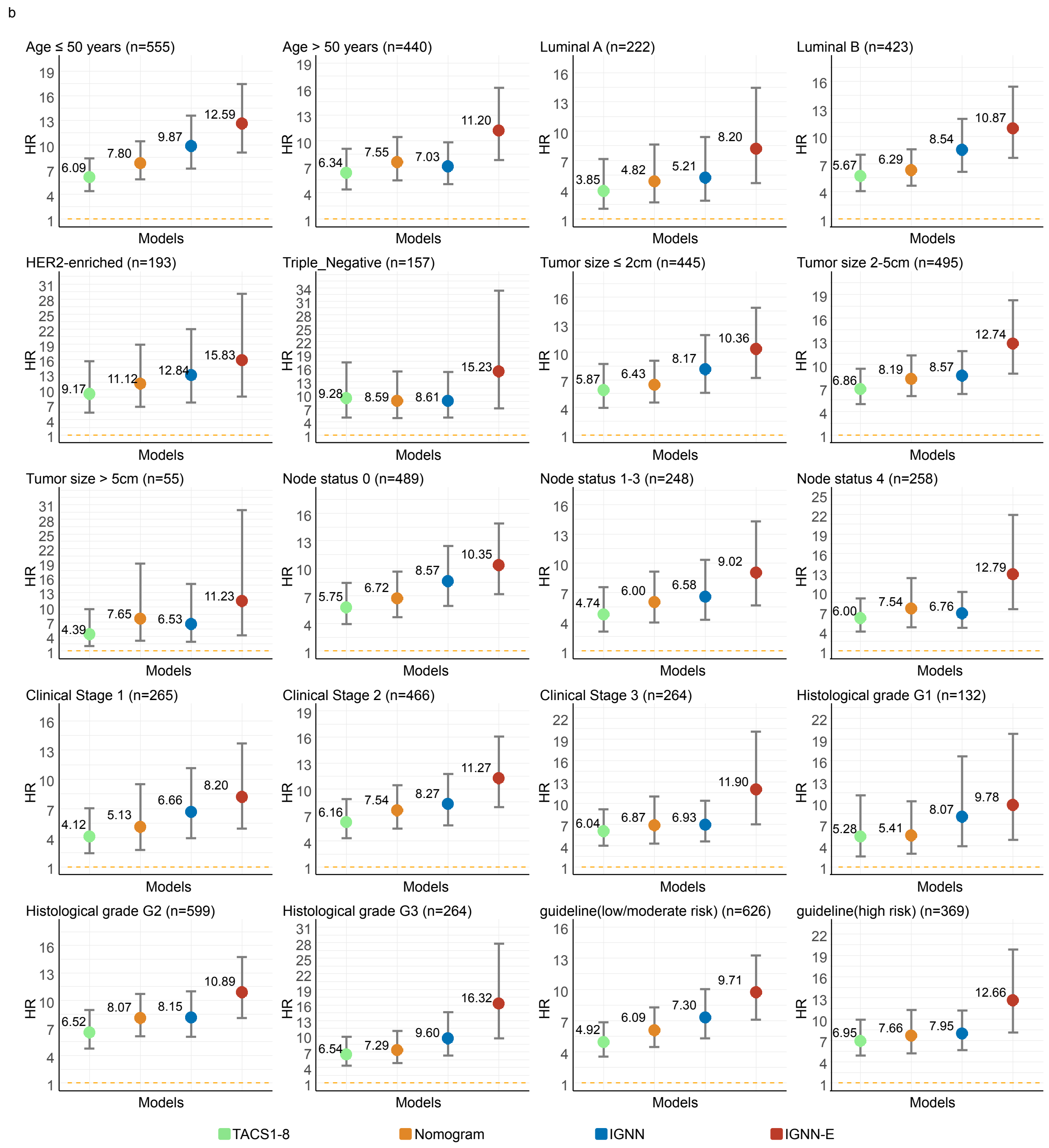

Supplementary Fig. 7. Performance of four prognostic models for different patient subgroups (*n* is number of patients within the specified subgroup). **(a)** Distribution of C-index for different prognostic models. For boxplots, middle line represents the median value, the upper and lower boundaries of boxes indicate 25th and 75th percentile, the whiskers reflect 1.5 times of interquartile range, the upper and lower tails indicate the maxima and minima, and single points indicate the outliers, respectively. A two-sided unpaired t-test was performed to determine significance**. (b)** Distribution of HR from Multivariate Cox proportional hazards regression analysis. For error bars, circles and upper/lower boundaries indicate mean value and 95% CIs, respectively. A two-sided log-rank test was performed to determine significance. HR, hazard ratio; C-index, concordance index; TACS (TACS1-8), tumor-associated collagen signatures; Nomogram, extended model of multivariate Cox proportional hazard regression; IGNN, intratumor graph neural network; IGNN-E, extended IGNN model with clinical information. Source data are provided as a Source Data file.

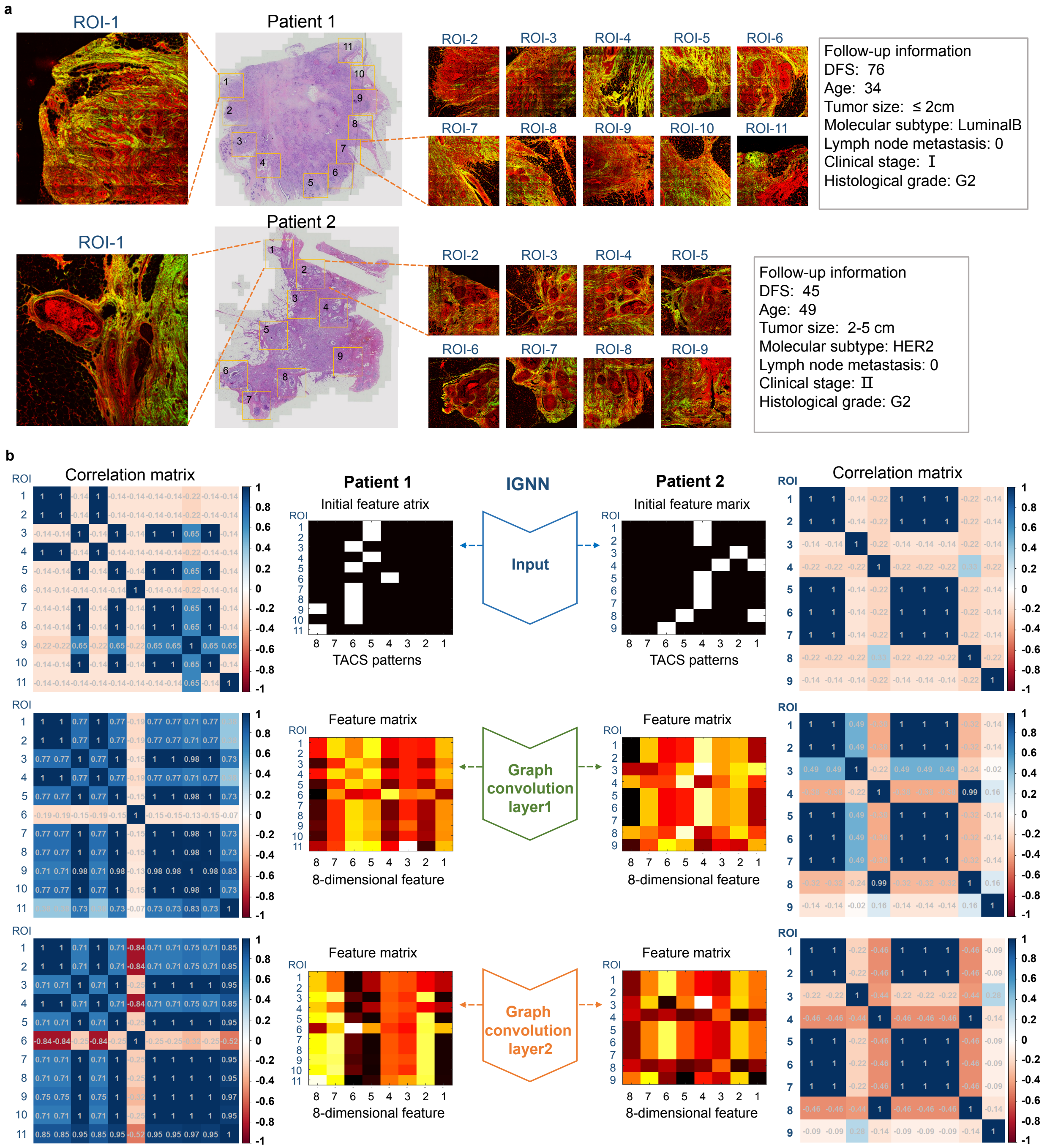

Supplementary Fig. 8. Pearson correlation analysis of graph node features. **(a)** Examples of two patients with different prognosis. **(b)** Visualization of the node state feature vectors corresponding to personalized MPM regions of interest (ROIs) and the heat maps of their Pearson correlation matrices. The correlation coefficients are annotated in the corresponding squares and indicated by a color scheme. For patient 1, collagen fibers show chaotic distribution (ROI$_{3,5,7\text{-}10}$ with TACS6) or penetrative distribution with obvious directionality (ROI $_{1,2,4}$ with TACS5) in multiple infiltrating frontier regions. The ROIs with TACS5 and ROIs with TACS6 are initially negatively correlated. The correlation increases significantly to ~0.9 when the corresponding state vectors are updated by the graph convolutional layers, consistent with our intuition that TACS6 and TACS5 promote tumor cell invasion. In contrast, more reticular distribution of collagen fibers (TACS4) in infiltrating frontier regions of patient 2 apparently blocks tumor cell invasion, as indicated by the low positive correlation between ROIs with TACS4 and ROIs with TACS5,6. ROI, regions of interest; DFS, disease-free survival; TACS (TACS1-8), tumor-associated collagen signatures; IGNN, intratumor graph neural network.

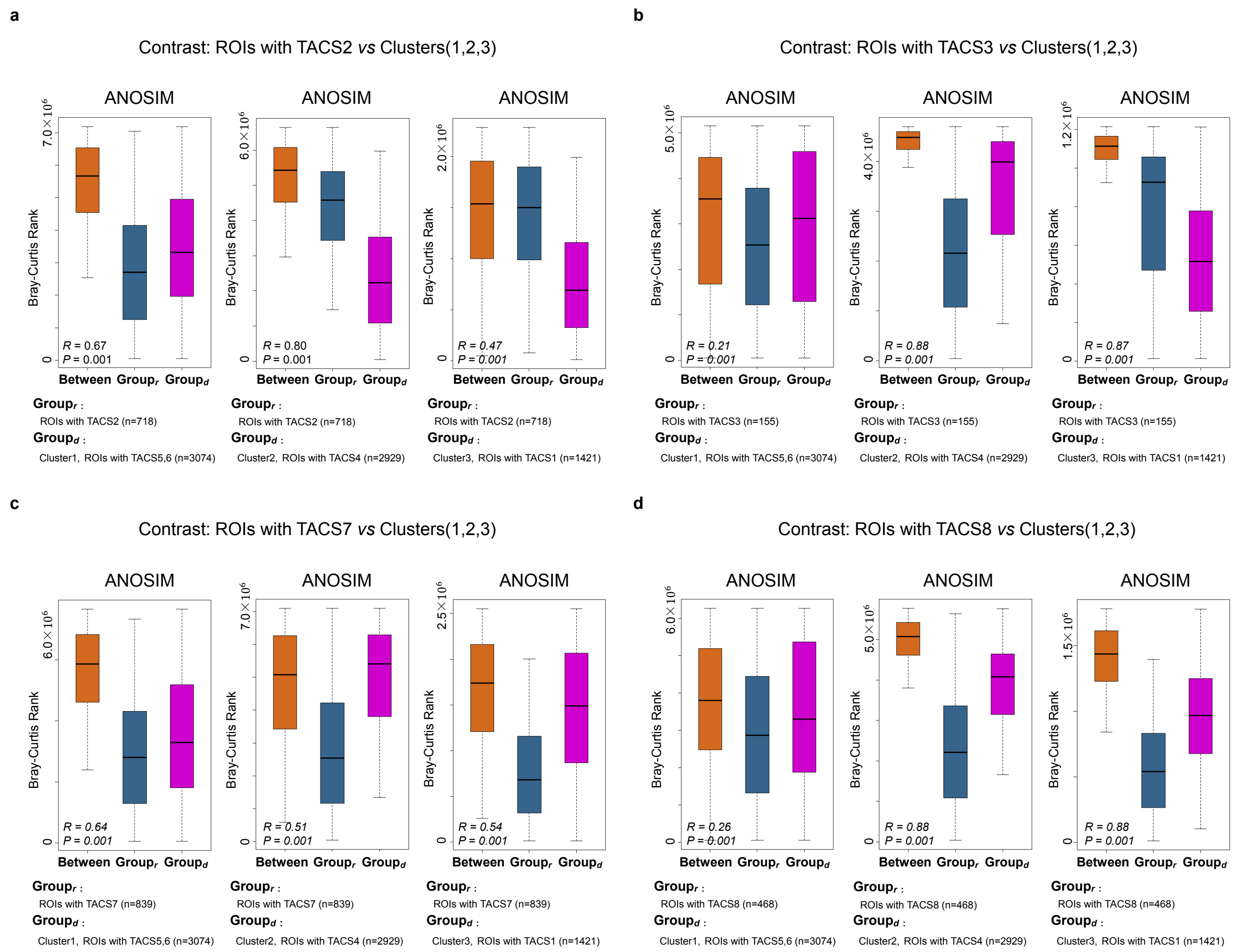

Supplementary Fig. 9. ANOSIM for assessing similarity between Group$_r$ consisting of ROIs with rare TACS features (TACS2,3,7,8) and Group$_d$ consisting of ROIs with dominant TACS features (TACS1,4,5,6). The boxplots marked with "Between", "Group$_r$", and "Group$_d$" show the Bray-Curtis rank distribution for sample pairs compared between Group$_r$ and Group$_d$, sample pairs within Group$_r$, and sample pairs within Group$_d$, respectively. In boxplots, middle line represent the median value, the upper and lower boundaries of boxes indicate 25th and 75th percentile, the whiskers reflect 1.5 times of interquartile range, the upper and lower tails indicate the maxima and minima; n is number of the ROIs with specified TACSs. Wilcoxon rank sum test was performed to compare the significant difference between groups without any adjustments for multiple comparisons. The statistic R ranges between the values −1.0 to 1.0, wherein $R < 0$ suggests that dissimilarities are greater within groups than between groups while $R > 0$ suggests greater dissimilarities between groups than within groups. The closer R value to 1.0, the greater the dissimilarities between samples from different groups. Observed R value over the null distribution is used to assess the significance of the statistic R, which was defined as $p <$ 0.05 from exact p values. **(a)** The lowest R value (R = 0.47) indicates the smallest dissimilarity between Cluster3 and the group consisting of ROI samples with TACS2; **(b)** The lowest R value (R = 0.21) indicates the smallest dissimilarity between Cluster1 and the group consisting of ROI samples with TACS3; **(c)** The lowest R value (R = 0.51) indicates the smallest dissimilarity between Cluster2 and the group consisting of ROI samples with TACS7; **(d)** The lowest R value (R = 0.26) indicates the smallest dissimilarity between Cluster1 and the group consisting of ROI samples with TACS8. ROI, regions of interest; TACS (TACS1-8), tumor-associated collagen signatures; ANOSIM, Analysis of similarities. Source data are provided as a Source Data file.

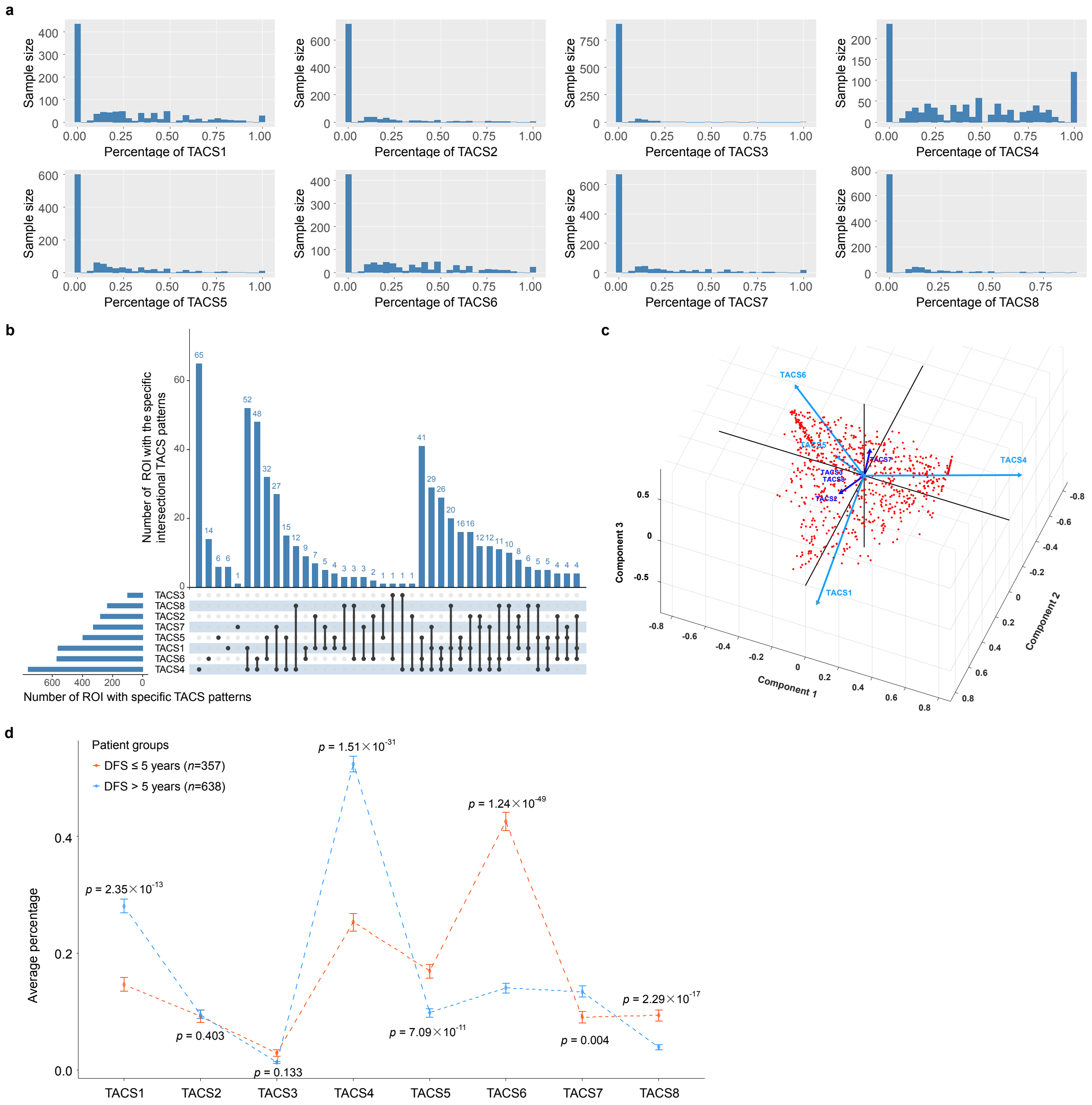

Supplementary Fig. 10. Distribution characteristics of the TACS1-8 in 995 patients. **(a)** Unbalanced size distribution of samples with different frequency of TACSs. **(b)** Size distribution of samples with multiple specific TACS patterns (multi-way intersection indicated by the multipoint link). **(c)** Principal component analysis (PCA) of distribution of 8 TACSs in samples, red dots indicate sample data points while arrows indicate TACS vectors defining the PCA space (the first three principal components were constructed using TACS6,4,1 associated vectors). For each given patient, P_{TACSi} (i = 1, 2, ... or 8) are calculated respectively as variables to indicate the percentage of TACSi present in all patient-related imaging regions, and PCA is used to visualize the characteristics of TACSs reflected by P_{TACSi}. The contribution of various TACSs to the individual principal component are different in the PCA representation space, among which TACS1,4,5,6 account for a greater proportion. **(d)** Differences in percentage of each TACS from patients with DFS over (blue) or less (red) than 5 years. The percentage of TACS were firstly calculated within each patient and then averaged across the patient groups (*n* is number of patients within specified patient groups). Error bars represent the mean values +/- SEM. A two-sided unpaired t-test was used to compare patient groups and determine significance from exact *p* values (significance was set at $p < 0.05$). ROI, regions of interest; TACS (TACS1-8), tumor-associated collagen signatures; DFS, disease-free survival. Source data are provided as a Source Data file.

Supplementary Table 1. Potential generalization of IGNN model to different technologies and biomarkers.

Technology	Sampling size (region of interest)	Biomarkers	Demonstrated breast cancer application	References
Multiphoton microscopy	~2.8 mm	TACS1-8	Improved prediction of prognosis by IGNN model	This study
H&E histology	0.2-10 mm	Histological types	Cancer diagnosis/prognosis	(1)
Immuno-histochemistry	0.2-10 mm	ER, PR, Her2, Ki67, etc.	Cancer diagnosis/prognosis	(2)
Multiregion omics	mm-scale	Molecular traits	Overall management	(3), (4)
Raman micro-spectroscopy*	mm-scale	Spectroscopic signatures	Intraoperative margin assessment	(5)
Infrared absorption micro-spectroscopy*	mm-scale	Spectroscopic signatures	Intraoperative diagnosis of benign vs. malignant tissue	(6)
Fluorescence micro-spectroscopy*	mm-scale	Spectroscopic signatures	Automated intraoperative detection of cancer	(7)
Mass micro-spectroscopy*	mm-scale	Spectroscopic signatures	Intraoperative margin assessment	(8)

Note: * - non-imaging spectroscopic technologies.

Supplementary Table 2. Details of IGNN model.

IGNN					
**Module Type**		**Layer**	**Layer Type**	**Filter Size**	**Feature Size**
		0	Input	-	N×8
	**Node feature embedding**	1	FConn	8×8	N×8
		2	SELU	-	N×8
**Graph convolution network module**	**Attention**	3-1	FConn	8×16	N*16
		3-2	SELU	-	N×16
		3-3	Norm	-	N×16
		3-4	Aggr	-	N×8
		3-5	Optional GRU	6×8×8	N×8
		4	Dropout	-	N×8
**Graph convolution network module**	**Attention**	5-1	FConn	8×16	N*16
		5-2	SELU	-	N×16
		5-3	Norm	-	N×16
		5-4	Aggr	-	N×8
		5-5	Optional GRU	6×8×8	N×8
		6	GPooling	-	8
**Fully connected network module**		7	FConn	8×32	32
		8	SELU	-	32
		9	FConn	32×32	32
		10	SELU	-	32
**Cox proportional hazards regression layer**		11	Survival regression	-	1

* **Aggr**, Aggregation; **Norm**, Normalization; **Gpooling**, Global mean pooling; **Fcon,** Fullyconnected; **SELU**, scaled exponential linear units. Dropout operations are used for training stages only.

Supplementary Table 3. Details of IGNN-E model.

IGNN-E					
**Module Type**		**Layer**	**Layer Type**	**Filter Size**	**Feature Size**
		0	Input	-	N×8
	**Embedding**	1	FConn	8×8	N×8
		2	SELU	-	N×8
**Graph convolution network module**	**Attention**	3-1	FConn	8×16	N*16
		3-2	SELU	-	N×16
		3-3	Aggr	-	N×8
		3-4	Optional GRU	6×8×8	N×8
		4	Dropout	-	N×8
**Graph convolution network module**	**Attention**	5-1	FConn	8×16	N*16
		5-2	SELU	-	N×16
		5-3	Norm	-	N×16
		5-4	Aggr	-	N×8
		5-5	Optional GRU	6×8×8	N×8
		6	GPooling	-	8
**Fully connected network module**		7	FConn	8×8	8
		8	SELU	-	8
		9	FConn	8×16	16
		10	SELU	-	16
		11	Input	-	9
**MLP**		12	FConn	9×16	16
		13	SELU	-	16
**Fusion layer**		14	FConn	32×32	32
		15	SELU	-	32
		16	Norm	-	32
		17	Dropout	-	32
**Cox proportional hazards regression layer**		18	Survival regression	-	1

* **Aggr**, Aggregation; **Norm**, Normalization; **Gpooling**, Global mean pooling; **Fcon,** Fullyconnected; **SELU**, scaled exponential linear units. Dropout operations are used for training stages only.

Supplementary Table 4. Four prognostic models with different biomarkers.

Prognostic model	Prognosticators	Method
**TACS1-8**	TACS (1-8) Patterns	Cox Ridge regression
**Nomogram**	TACS (1-8) Patterns<br>+ Age + Molecular subtype + Tumor size + Lymph node metastasis + Clinical stage + Histological grade + Chemotherapy + Radiation Therapy	Multivariate Cox proportional hazard regression
**IGNN**	TACS associated graph structure data	Graph neural network
**IGNN-E**	TACS associated graph structure data + Age + Molecular subtype + Tumor size + Lymph node metastasis + Clinical stage + Histological grade + Chemotherapy + Radiation Therapy	Graph neural network combined with MLP

* **TACS (TACS1-8)**, tumor-associated collagen signatures; **Nomogram**, extended model of multivariate Cox proportional hazard regression; **IGNN**, intratumor graph neural network; **IGNN-E**, extended model by incorporating clinical information to the basic IGNN model.

Supplementary Table 5. Performance to predict 5-year DFS rates of patients in training cohort by four prognostic models.

Pre-validation						
**Models**	**Training cohort (n=731)**					
	**AUC_{risk}**	**Sens**	**Spec**	**PPV**	**NPV**	**Acc**
**TACS1-8**	0.766	0.759	0.770	0.647	0.852	0.766
**IGNN**	0.791	0.824	0.757	0.654	0.886	0.781
**Nomogram**	0.775	0.793	0.757	0.645	0.868	0.770
**IGNN-E**	0.819	0.847	0.792	0.693	0.903	0.811

***AUC_{risk}**, area under the receiver operating characteristic curve according to the prognostic risk; **Sens**, sensitivity; **Spec**, specificity; **PPV**, positive predict value; **NPV**, negative predict value; **Acc**, accuracy; **TACS (TACS1-8)**, tumor-associated collagen signatures; **Nomogram**, extended model of multivariate Cox proportional hazard regression; **IGNN**, intratumor graph neural network; **IGNN-E**, extended model by incorporating clinical information to the basic IGNN model.

Supplementary Table 6. Multivariate Cox proportional hazards regression analysis including IGNN score and clinicopathological factors for DFS in training and validation cohorts.

Factors	Training cohort (n = 731)			Validation cohort (n = 264)		
	**Exp (Coef) (95%CI)**	**Se (Coef)**	***P* Value**	**Exp (Coef) (95%CI)**	**Se (Coef)**	***P* Value**
**IGNN score**						
Low risk	Reference			Reference		
High risk	8.654 (6.465 - 11.585)	0.149	$< 2.00\times10^{-16}$	5.882 (3.801 – 9.101)	0.223	1.80×10^{-15}
**Molecular subtype**						
Luminal A	Reference			Reference		
Luminal B	1.921 (1.289 - 2.862)	0.204	0.0013	2.719 (1.469 - 5.025)	0.314	0.0015
HER2-enriched	2.019 (1.291 - 3.158)	0.228	0.0021	3.175 (1.568 – 6.435)	0.360	0.0013
Triple-negative	2.584 (1.638 – 4.077)	0.233	4.50×10^{-5}	3.528 (1.719 – 7.244)	0.367	0.0006
**Tumor size**						
≤2cm	Reference			Reference		
2-5cm	1.609 (1.117 - 2.318)	0.186	0.0107	1.204 (0.750 - 1.932)	0.241	0.4415
≥5cm	2.291 (1.390 - 3.778)	0.255	0.0012	1.381 (0.312 - 6.111)	0.759	0.6708
**Lymphnode metastasis**						
0	Reference			Reference		
1-3	1.484 (1.005 – 2.190)	0.199	0.0472	1.754 (0.804 - 3.832)	0.398	0.1583
≥4	3.656 (1.756 – 7.614)	0.374	0.0005	1.212 (0.127 – 11.531)	1.149	0.8674
**Clinical stage**						
I	Reference			Reference		
II	0.703 (0.407 - 1.216)	0.279	0.2080	0.580 (0.233 – 1.442)	0.465	0.2414
III	0.454 (0.187 - 1.097)	0.451	0.0795	1.460 (0.144 – 14.82)	1.183	0.7490
**Histological grade**						
G1	Reference			Reference		
G2	1.125 (0.763 - 1.660)	0.198	0.5513	1.091 (0.327 - 3.637)	0.615	0.8871
G3	1.472 (0.975 - 2.225)	0.211	0.0661	1.882 (0.526 - 6.730)	0.650	0.3308
**Chemotherapy**						
Yes	Reference			Reference		
No	0.625 (0.429 - 0.909)	0.192	0.0141	0.696 (0.327 - 1.481)	0.385	0.3470
**Radiationtherapy**						
Yes	Reference			Reference		
No	0.860 (0.651 - 1.137)	0.142	0.2893	1.623 (0.955 – 2.759)	0.271	0.0737
**Age**						
≤50	Reference			Reference		
>50	0.910 (0.711 – 1.165)	0.126	0.4540	1.042 (0.697 - 1.563)	0.205	0.8404

A two-sided wald-test was performed to determine significance without any adjustments for multiple comparisons and the significance was defined as $p < 0.05$.

* **HER2**, human epidermal growth factor receptor 2; **IGNN**, intratumor graph neural network; **Exp(coef)**, exponentiated coefficients, also known as hazard ratios; **Se(coef)**, standard error of hazard ratios**.**

Supplementary Table 6 (continued). Multivariate Cox proportional hazards regression analysis including TACS score and clinicopathological factors for DFS in training and validation cohorts.

Factors	Training cohort (n = 731)			Validation cohort (n = 264)		
	**Exp (Coef) (95%CI)**	**Se (Coef)**	***P* Value**	**Exp (Coef) (95%CI)**	**Se (Coef)**	**P Value**
**TACS1-8 score**						
Low risk	Reference			Reference		
High risk	6.378 (4.729 - 8.603)	0.153	$< 2.00\times10^{-16}$	5.020 (3.220 - 7.834)	0.227	1.10×10^{-12}
**Molecular subtype**						
Luminal A	Reference			Reference		
Luminal B	1.842 (1.240 - 2.735)	0.202	0.0025	2.725 (1.481 - 5.014)	0.311	0.0013
HER2-enriched	2.012 (1.288 – 3.145)	0.228	0.0022	3.536 (1.743 – 7.180)	0.361	0.0005
Triple-negative	2.619 (1.653 – 4.150)	0.235	4.10×10^{-5}	4.132 (2.009 – 8.504)	0.368	0.0001
**Tumor size**						
≤2cm	Reference			Reference		
2-5cm	1.552 (1.078 – 2.233)	0.186	0.0180	1.390 (0.867 - 2.233)	0.240	0.1711
≥5cm	2.483 (1.507 – 4.090)	0.255	0.0004	1.600 (0.363 – 7.062)	0.757	0.5349
**Lymphnode metastasis**						
0	Reference			Reference		
1-3	1.454 (0.988 - 2.140)	0.197	0.0574	2.073 (0.963 - 4.457)	0.391	0.0622
≥4	4.311 (2.058 - 9.032)	0.377	0.0001	1.384 (0.148 - 12.902)	1.139	0.7754
**Clinical stage**						
I	Reference			Reference		
II	0.841 (0.488 - 1.450)	0.278	0.5337	0.551 (0.224 - 1.359)	0.460	0.1959
III	0.483 (0.198 - 1.178	0.455	0.1095	1.510 (0.150 - 15.190)	1.178	0.7266
**Histological grade**						
G1	Reference			Reference		
G2	1.209 (0.821 – 1.779)	0.197	0.3362	0.906 (0.271 - 3.020)	0.615	0.8720
G3	1.439 (0.951 - 2.178)	0.212	0.0854	1.592 (0.445 - 5.691)	0.650	0.4745
**Chemotherapy**						
Yes	Reference			Reference		
No	0.588 (0.402 - 0.859)	0.194	0.0061	0.620 (0.293 - 1.311)	0.382	0.2101
**Radiationtherapy**						
Yes	Reference			Reference		
No	0.896 (0.678 - 1.183)	0.142	0.4392	1.634 (0.970 - 2.745)	0.266	0.0650
**Age**						
≤50	Reference			Reference		
>50	0.955 (0.748 - 1.221)	0.125	0.7144	1.125 (0.751 - 1.693)	0.206	0.5681

A two-sided wald-test was performed to determine significance without any adjustments for multiple comparisons, and the significance was defined as $p < 0.05$.

* **HER2**, human epidermal growth factor receptor 2; **TACS (TACS1-8)**, tumor-associated collagen signatures; **Exp(coef)**, exponentiated coefficients, also known as hazard ratios; **Se(coef)**, standard error of hazard ratios.

Supplementary Table 7. Performance to predict 5-year DFS rates of patients in training and validation cohorts by four prognostic models.

External validation												
**Models**	**Training cohort (n=731)**						**Validation cohort (n=264)**					
	**AUC**	**Sens**	**Spec**	**PPV**	**NPV**	**Acc**	**AUC**	**Sens**	**Spec**	**PPV**	**NPV**	**Acc**
**TACS1-8**	0.834	0.812	0.749	0.642	0.878	0.771	0.803	0.760	0.714	0.603	0.839	0.731
**IGNN**	0.868	0.782	0.855	0.750	0.876	0.829	0.826	0.755	0.827	0.713	0.853	0.799
**Nomogram**	0.877	0.720	0.877	0.764	0.850	0.821	0.850	0.646	0.887	0.765	0.814	0.799
**IGNN-E**	0.913	0.828	0.881	0.794	0.902	0.862	0.877	0.750	0.887	0.791	0.861	0.837

***AUC**, area under the receiver operating characteristic curve according to the prognostic score; **Sens**, sensitivity; **Spec**, specificity; **PPV**, positive predict value; **NPV**, negative predict value; **Acc**, accuracy; **TACS (TACS1-8)**, tumor-associated collagen signatures; **Nomogram**, extended model of multivariate Cox proportional hazard regression; **IGNN**, intratumor graph neural network; **IGNN-E**, extended model by incorporating clinical information to the basic IGNN model.

Supplementary Table 8. Survival characteristics of patient subgroups with different prognostic biomarkers.

Subgroups	Clinicopathological factors	All cohort (n=995)		
		Number of patients (%)	DFS ≤ 5 years	DFS > 5 years
1	**Age** (≤ 50 years)	555 (55.8%)	186	369
2	**Age** (> 50 years)	440 (44.2%)	171	269
3	**Molecular subtype** (Luminal A)	222 (22.3%)	42	180
4	**Molecular subtype** (Luminal B)	423 (42.5%)	168	255
5	**Molecular subtype** (HER2 enriched)	193 (19.4%)	75	118
6	**Molecular subtype** (Triple Negative)	157 (15.8%)	72	85
7	**Tumor size** (≤ 2cm)	445 (44.7%)	119	326
8	**Tumor size** (2-5cm)	495 (49.8%)	202	293
9	**Tumor size** (> 5cm)	55 (5.5%)	36	19
10	**Lymph node metastasis** (0)	489 (49.1%)	109	380
11	**Lymph node metastasis** (1-3)	248 (24.9%)	91	157
12	**Lymph node metastasis** (4)	258 (25.9%)	157	101
13	**Clinical stage** (Ⅰ)	265 (26.6%)	49	216
14	**Clinical stage** (Ⅱ)	466 (46.9%)	149	317
15	**Clinical stage** (Ⅲ)	264 (26.5%)	159	105
16	**Histological grade** (G1)	132 (13.3%)	34	98
17	**Histological grade** (G2)	599 (60.2%)	202	397
18	**Histological grade** (G3)	264 (26.5%)	121	143
19	**Guideline** (low/moderate risk)	626 (62.9%)	150	476
20	**Guideline** (high risk)	369 (37.1%)	207	162

Supplementary Table 9. TACS-containing ROIs from all patients under study (n = 995).

Total number of ROIs	ROIs with 1 TACS features	ROIs with 2 TACS features	ROIs with 3 TACS features	ROIs with ≥ 4 TACS features
7424	5357	1781	261	25
percentage	72.2%	24.0%	3.51%	0.34%

* **ROI**, regions of interest; **TACS (TACS1-8)**, tumor-associated collagen signatures.

Supplementary Table 10. Effect of random removal of 20% of ROIs on IGNN prognosis of individual patients in the validation cohort with >6 ROIs (n = 212).

Trial	Consistency (high risk)	Consistency (low risk)
1	0.963	0.969
2	0.963	0.977
3	0.951	0.969
4	0.975	0.969
5	0.975	0.969
6	0.975	0.969
7	0.975	0.969
8	0.963	0.977
9	0.951	0.969
10	0.951	0.969
11	0.951	0.977
12	0.963	0.977
13	0.963	0.969
14	0.963	0.969
15	0.963	0.969
16	0.963	0.969
17	0.963	0.969
18	0.951	0.969
19	0.975	0.969
20	0.963	0.969

Note: To evaluate the impact of potential variability in the number and distribution of selected ROIs on the risk stratification ability of IGNN model, we randomly removed 20% ROIs from the sample and repeat the prediction using the well-trained IGNN model. The new prediction remains highly consistent with the original prediction based on all ROIs.

References:

1. Weigelt, B., Geyer, F. C. & Reis-Filho, J. S. Histological types of breast cancer: how special are they? *Mol Oncol*. **4**,192-208 (2010).
2. Zaha D. C. Significance of immunohistochemistry in breast cancer. *World J. Clin. Oncol.* **5**, 382-392 (2014).
3. Yates, L., Gerstung, M., Knappskog, S. et al. Subclonal diversification of primary breast cancer revealed by multiregion sequencing. *Nat. Med*. **21**, 751-759 (2015).
4. Karczewski, K., Snyder, M. Integrative omics for health and disease. *Nat. Rev. Genet.* **19**, 299-310 (2018).
5. Haka, A. S., Volynskaya, Z., Gardecki, J. A., Nazemi, J., Lyons, J., Hicks, D., Fitzmaurice, M., Dasari, R. R., Crowe, J. P., & Feld, M. S. *In vivo* margin assessment during partial mastectomy breast surgery using raman spectroscopy. *Cancer Res*. **66**, 3317-3322 (2006).
6. Tian, P., Zhang, W., Zhao, H., Lei, Y., Cui, L., Wang, W., Li, Q., Zhu, Q., Zhang, Y., & Xu, Z. Intraoperative diagnosis of benign and malignant breast tissues by fourier transform infrared spectroscopy and support vector machine classification. *Int. J. Clin. Exp. Med.* **8**, 972-981 (2015).
7. Phipps, J. E., Gorpas, D., Unger, J., Darrow, M., Bold, R. J., & Marcu, L. Automated detection of breast cancer in resected specimens with fluorescence lifetime imaging. *Phys. Med. Biol.* **63**, 015003 (2017).
8. Balog, J., Sasi-Szabó, L., Kinross, J., Lewis, M. R., Muirhead, L. J., Veselkov, K., Mirnezami, R., Dezső, B., Damjanovich, L., Darzi, A., Nicholson, J. K., & Takáts, Z. Intraoperative tissue identification using rapid evaporative ionization mass spectrometry. *Sci. Transl. Med.* **5**, 194ra93 (2013).
