# Supplementary material for: Intratumor graph neural network recovers hidden prognostic value of multi-biomarker spatial heterogeneity
Source: Nat Commun. 2022 Jul 22;13:4250. doi: 10.1038/s41467-022-31771-w (PMC9307796; doi:10.1038/s41467-022-31771-w)
Supplement: Supplementary file 3 — Reporting Summary [file 41467_2022_31771_MOESM3_ESM.pdf]

nature portfolio

Corresponding author(s): Lianhuang Li, Jianxin Chen, Haohua Tu

Last updated by author(s): May 29, 2022

Reporting Summary

Nature Portfolio wishes to improve the reproducibility of the work that we publish. This form provides structure for consistency and transparency in reporting. For further information on Nature Portfolio policies, see our Editorial Policies and the Editorial Policy Checklist.

Statistics

For all statistical analyses, confirm that the following items are present in the figure legend, table legend, main text, or Methods section.

n/a	Confirmed	
☐	☒	The exact sample size (n) for each experimental group/condition, given as a discrete number and unit of measurement
☐	☒	A statement on whether measurements were taken from distinct samples or whether the same sample was measured repeatedly
☐	☒	The statistical test(s) used AND whether they are one- or two-sided<br>*Only common tests should be described solely by name; describe more complex techniques in the Methods section.*
☐	☒	A description of all covariates tested
☐	☒	A description of any assumptions or corrections, such as tests of normality and adjustment for multiple comparisons
☐	☒	A full description of the statistical parameters including central tendency (e.g. means) or other basic estimates (e.g. regression coefficient) AND variation (e.g. standard deviation) or associated estimates of uncertainty (e.g. confidence intervals)
☐	☒	For null hypothesis testing, the test statistic (e.g. F, t, r) with confidence intervals, effect sizes, degrees of freedom and P value noted<br>*Give P values as exact values whenever suitable.*
☒	☐	For Bayesian analysis, information on the choice of priors and Markov chain Monte Carlo settings
☐	☒	For hierarchical and complex designs, identification of the appropriate level for tests and full reporting of outcomes
☒	☐	Estimates of effect sizes (e.g. Cohen's d, Pearson's r), indicating how they were calculated

Our web collection on statistics for biologists contains articles on many of the points above.

Software and code

Policy information about availability of computer code

Data collection	MPM images were scanned via an imaging system that was built on a commercially laser scanning microscope platform (LSM 880 Zeiss, Germany) using a mode-locked femtosecond Ti:Sapphire laser (Chameleon ultra?, Coherent, America).
Data analysis	R 3.6.0 (http://www.R-project.org)

For manuscripts utilizing custom algorithms or software that are central to the research but not yet described in published literature, software must be made available to editors and reviewers. We strongly encourage code deposition in a community repository (e.g. GitHub). See the Nature Portfolio guidelines for submitting code & software for further information.

Data

Policy information about availability of data

All manuscripts must include a data availability statement. This statement should provide the following information, where applicable:

- Accession codes, unique identifiers, or web links for publicly available datasets
- A description of any restrictions on data availability
- For clinical datasets or third party data, please ensure that the statement adheres to our policy

The main figures and tables containing raw data can be found in the Source Data File. The imaging data can be provided upon request to the corresponding author.

Field-specific reporting

Please select the one below that is the best fit for your research. If you are not sure, read the appropriate sections before making your selection.

☒ Life sciences ☐ Behavioural & social sciences ☐ Ecological, evolutionary & environmental sciences

For a reference copy of the document with all sections, see nature.com/documents/nr-reporting-summary-flat.pdf

Life sciences study design

All studies must disclose on these points even when the disclosure is negative.

Sample size	The inclusion criteria were: (i) patients had pathologically confirmed invasive breast cancer (IBC) without distant metastasis and underwent surgical resection; and (ii) clinicopathological characteristics and follow-up information were complete. Finally, 995 patients were enrolled for analysis. Thus, the sample size of the study, about 1000, was sufficiently sized to support the research objectives.
Data exclusions	The exclusion criteria were: (i) patients were treated with preoperative therapy (neoadjuvant chemotherapy and radiotherapy); and (ii) patients were suffered from other tumor disease at the same time; and(iii) some samples were not suitable for analysis due to the damage and tumor-free of sections.
Replication	The study design included separate training and validation data. The validation data was not used to determine model estimates. Models were verified and replicated on validation cohorts.
Randomization	Not applicable
Blinding	MPM images were visually examined by three independent reviewers who were blind to the final pathological outcomes.

Reporting for specific materials, systems and methods

We require information from authors about some types of materials, experimental systems and methods used in many studies. Here, indicate whether each material, system or method listed is relevant to your study. If you are not sure if a list item applies to your research, read the appropriate section before selecting a response.

Materials & experimental systems

n/a	Involved in the study
☒	☐ Antibodies
☒	☐ Eukaryotic cell lines
☒	☐ Palaeontology and archaeology
☒	☐ Animals and other organisms
☒	☐ Human research participants
☒	☐ Clinical data
☒	☐ Dual use research of concern

Methods

n/a	Involved in the study
☒	☐ ChIP-seq
☒	☐ Flow cytometry
☒	☐ MRI-based neuroimaging
